# Estimating the association of hypertensive disorders of pregnancy with offspring kidney function from adolescence into young adulthood

**DOI:** 10.1007/s00467-026-07273-y

**Published:** 2026-03-31

**Authors:** Jo A. Kelly, Mark C. Chappell, Elizabeth T. Jensen, Keia R. Sanderson, Christopher L. Schaich, Hossam A. Shaltout, Andrew M. South

**Affiliations:** 1 Wake Forest University School of Medicine, Winston-Salem, NC 27157 USA; 2 Department of Surgery, Section of Hypertension and Vascular Research, Wake Forest University School of Medicine, Winston-Salem, NC 27157 USA; 3 Division of Public Health Sciences, Department of Epidemiology and Prevention, Wake Forest University School of Medicine, Winston-Salem, NC 27157 USA; 4 Department of Pediatrics, Division of Pediatric Nephrology, University of North Carolina, Chapel Hill, NC 27599 USA; 5 Department of Obstetrics and Gynecology, Wake Forest University School of Medicine, Winston Salem, NC 27157 USA; 6 Department of Pediatrics, Section of Nephrology, Atrium Health Levine Children’s Brenner Children’s Hospital, Wake Forest University School of Medicine, Winston Salem, NC 27157 USA

**Keywords:** Pediatric nephrology, Pediatrics, Organogenesis, Pregnancy complications, Causal inference, Directed acyclic graphs, Causal mediation analysis

## Abstract

**Background:**

To estimate the association of hypertensive disorders of pregnancy (HDP) with offspring kidney function in adolescence and young adulthood in individuals born preterm with very low birth weight.

**Methods:**

Secondary analysis of data from a prospective cohort study of individuals born preterm with very low or extremely low birth weight (< 1500 g). The 213 participants were assessed at 14–15 and 19–23 years of age. Outcomes were estimated glomerular filtration rate (eGFR) and first-morning urine albumin-to-creatinine ratio (ACR). We estimated the relationships with multivariable generalized linear models informed by directed acyclic graphs. Causal mediation analysis was also performed to evaluate if adolescent kidney function indirectly mediated the effect of HDP on young adulthood kidney function.

**Results:**

Thirty-seven percent (*n* = 79) were exposed to HDP. Kidney function in adolescence was associated with kidney function in young adulthood (eGFR adjusted *β* 0.48 mL/min/1.73 m^2^, 95% CL 0.21–0.75; ACR adjusted *β* 0.19 mg/g, 95% CL 0.15–0.24). There was no significant association between HDP and kidney function in both adolescence and young adulthood. There was also no statistically significant causal mediation effect of HDP on young adulthood kidney function indirectly through adolescent kidney function.

**Conclusions:**

We found no significant association between HDP and offspring kidney function in adolescence and young adulthood and no evidence of mediation through adolescent kidney function. Next steps in this research may look to investigate the relationship of severity of prematurity on offspring kidney function, as HDPs are a factor in premature births and the degree of prematurity.

**Graphical Abstract:**

**Figure:**
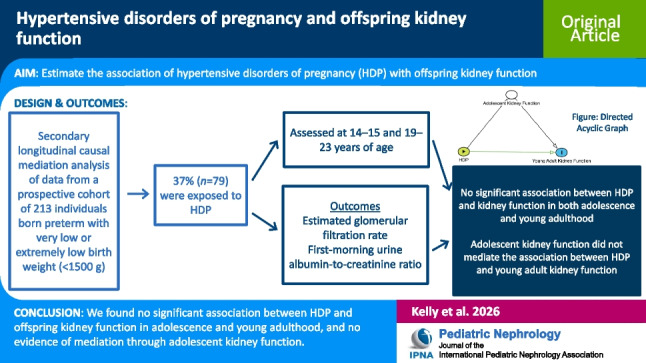
A higher resolution version of the Graphical abstract is available as Supplementary information.

**Supplementary Information:**

The online version contains supplementary material available at 10.1007/s00467-026-07273-y.

## Introduction

Hypertensive disorders of pregnancy (HDPs) are associated with significant health complications for both the mother and offspring [1]. These disorders include chronic hypertension (diagnosed < 20 weeks’ gestation), gestational hypertension (≥ 20 weeks’ gestation), and preeclampsia (evidence of target organ dysfunction most notably proteinuria)—including that superimposed on chronic hypertension—and eclampsia (preeclampsia but with acute life-threatening organ damage) [1, 2]. Several studies have shown that youth exposed to preeclampsia in utero are more likely to be hospitalized during their lifetime up to 27 years old, along with having a higher blood pressure and body mass index in childhood through adolescence [1, 3]. Preterm birth is associated with a higher risk of adverse health outcomes and an increased risk of mortality [4, 5].

While there have been several extensive investigations into premature offspring’s health outcomes, especially cardiovascular outcomes, little research has focused on the change in kidney function over time into adulthood. The goal of this analysis is to better understand the association of HDP with future offspring kidney function in adolescence and young adulthood in a well-characterized long-term birth cohort of individuals born preterm with very low or extremely low birth weight. Our aim is to estimate the direct effect of HDP on offspring kidney function in young adulthood and the indirect effect mediated through adolescent kidney function.

## Methods

### Study design

This is a secondary longitudinal analysis of data collected in a long-term prospective cohort study of individuals born with very low or extremely low birth weight (birth weight < 1500 g), the Prenatal Events-Postnatal Consequences (PEPC) study. Participants were initially recruited at age 14 years (PEPC1) and were assessed over a series of three study visits. Participants were then recruited into the second iteration of the study starting at age 18 years (PEPC2) and were assessed over a series of two study visits. Due to sample size attrition between PEPC1 and PEPC2, 28 individuals who were not enrolled in the PEPC1 study but met inclusion criteria and lacked exclusion criteria were also recruited and enrolled in the PEPC2 study. The institutional review board approved the study, and written informed consent and assent were obtained from parents or legal guardians and participants at PEPC1 and PEPC2. Further details on the PEPC study design have been previously published [5].

### Study population

**Fig. 1:**
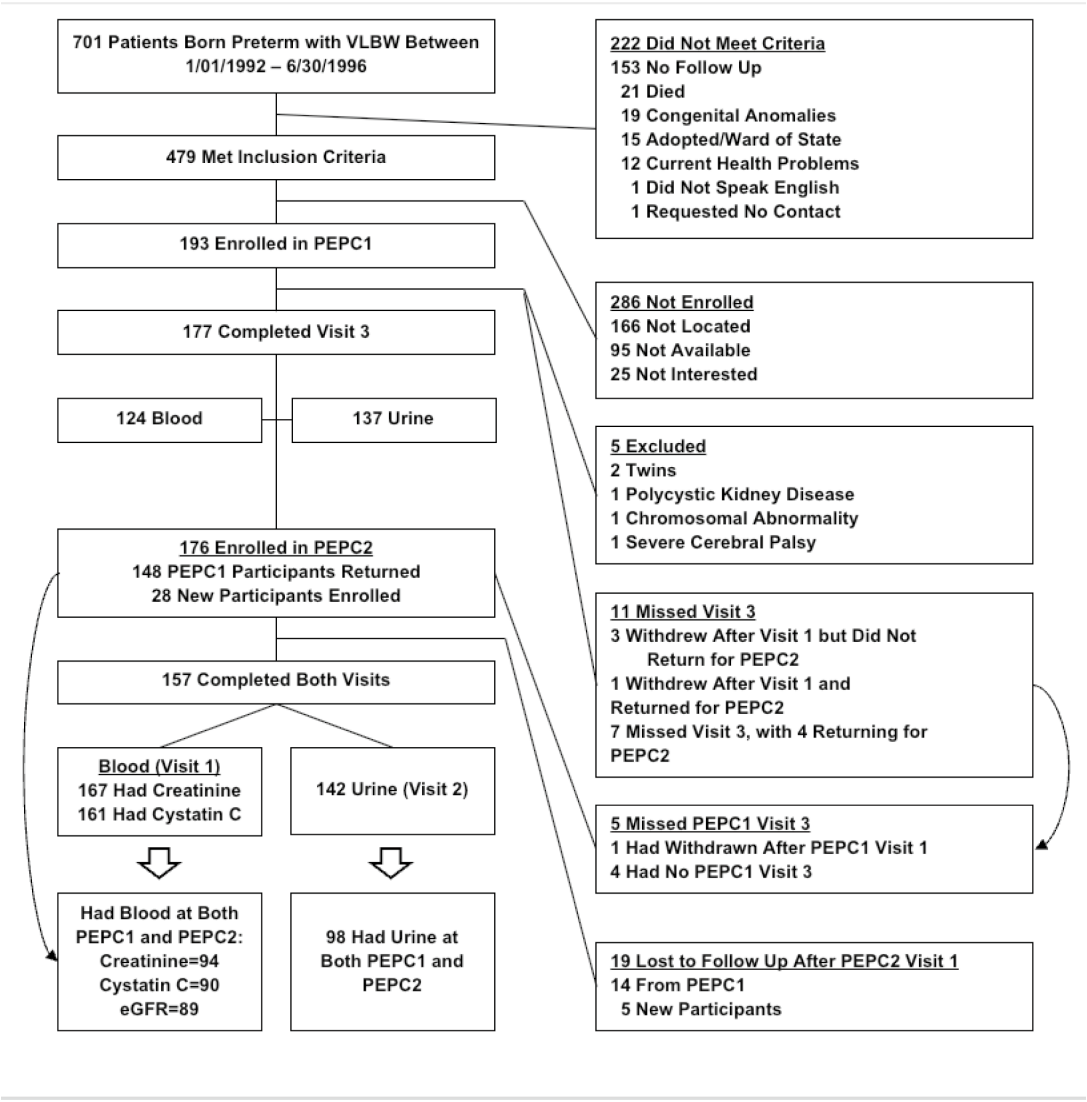
Exclusion criteria flowchart for PEPC1 and PEPC2. Two hundred thirteen participants used in this study are composed of the 193 enrolled in PEPC1 (except the 5 excluded and 3 that withdrew) and the 28 new participants in PEPC2. eGFR, estimated glomerular filtration rate; PEPC, Prenatal Events-Postnatal Consequences; VLBW, very low or extremely low birth weight

Inclusion criteria were singleton births at Forsyth Medical Center in Winston Salem, North Carolina, from January 1, 1992, to June 30, 1996, birth weight < 1500 g, follow-up data through 1-year corrected age, successful contact at 14 years old for PEPC1, and successful contact at 18–23 years old for PEPC2. Exclusion criteria were significant congenital malformations, primary health conditions in adolescence or young adulthood, and inability to complete study procedures. For the primary analysis, we excluded participants lacking blood and urine samples for kidney function analysis at PEPC1 and PEPC2 (Fig. 1).

### Study data and procedures

Study staff retrospectively recorded antenatal and infant data from health records and research databases. The study staff administered questionnaires to parents/legal guardians at the first study visit in PEPC1 and again in PEPC2 for new participants. The original study design collected race only as self-reported Black or non-Black. In this analysis, we conceptualized race as a social construct, not as a proxy for biological (e.g., inherited) traits, ancestry, socioeconomic status (e.g., social risk factors), or racism.

For descriptive purposes, we classified participants by gestational age at birth per guidelines: (i) moderately preterm for gestational age ≥ 32 weeks to < 34 weeks, (ii) very preterm for ≥ 28 weeks to < 32 weeks, and (iii) extremely preterm for < 28 weeks [6]. For descriptive purposes, we classified participants’ birth weight per guidelines: (i) very low birth weight for birth weight ≥ 1000 to < 1500 g and (ii) extremely low birth weight for birth weight < 1000 g [7].

In PEPC1, participants’ blood samples and first-morning urine samples (collected at home and brought directly to the study visit) were obtained at the third study visit; these collections were optional in the study design. Serum creatinine was measured to estimate the glomerular filtration rate (eGFR). eGFR was calculated using the original Schwartz equation *k* × height (cm)/serum creatinine (mg/dL), with *k* = 0.55 for females and *k* = 0.7 for males [5]. We chose this equation because it is validated in a general pediatric population lacking known chronic kidney disease and thus is less likely to underestimate kidney function [8]. Urinary albumin and creatinine were measured from the first-morning urine sample. Those values were then used to calculate the albumin-to-creatinine ratio (ACR), and albuminuria was defined as a ratio > 30 mg/g [9]. The creatinine was analyzed using a modified Jaffe assay traceable to isotope mass spectrometry, which was later updated during the study period and a correction value of 1.06 was applied to the relevant original values [5].

In PEPC2, participants’ blood samples were obtained at visit 1, while the first-morning urine sample was collected at home and brought by the participant to visit 2. Serum and urine creatinine were measured with a modified Jaffe assay traceable to isotope mass spectrometry, and serum cystatin C was measured with an ELISA [9]. eGFR was estimated using the 2021 CKD-EPI equation for creatinine and cystatin C. The ACR was calculated, and albuminuria was defined the same as mentioned above in PEPC1. Our exposure was HDP, which included chronic hypertension, gestational hypertension, or preeclampsia; our original study design’s data collection methods did not reliably distinguish between these conditions, and thus, we did not analyze them individually due to concern for measurement error [10]. These conditions were reported via questionnaires. Our outcomes were eGFR, ACR, and albuminuria at PEPC2. Our mediators were eGFR, ACR, and albuminuria at PEPC1.

### Statistical analysis

To summarize the data, we utilized measures of central tendency, including mean with SD and median with interquartile range. We also applied the chi-squared test, Fisher exact test, *t*-test, and Wilcoxon rank-sum test for between-group comparisons in Table 1, as indicated by whether test assumptions were met. A two-sided alpha level of less than 0.05 was indicative of statistical significance. SAS Enterprise Guide software version 7.1 for Windows (SAS Institute Inc., Cary, NC) and R 4.2.3 (2023) were used for all data analysis.

**Table 1:** Participant characteristics at adolescence and young adulthood by exposure to hypertensive disorders of pregnancy

	Study population *N* = 213	HDP *n* = 79 (37%)	No HDP *n* = 134 (63%)
Female	113 (53%)	44 (56%)	69 (51%)
Black	89 (42%)	28 (35%)	61 (46%)
Gestational age at birth (weeks)	27.9 (2.6)	29.0 (2.7)^*^	27.2 (2.4)
Birth weight (g)	1060 (268)	1090 (266)	1043 (268)
Age at PEPC1 (years)	14.6 (0.3)	14.6 (0.3)	14.6 (0.3)
Age at PEPC2 (years)	19.7 (0.8)	19.6 (0.8)	19.7 (0.9)

*HDPs* hypertensive disorders of pregnancy

^*^p value < 0.05 by *t*-test

We first used bivariate and multivariable generalized linear models to estimate the associations of our exposures with our outcomes. No participants at PEPC1 or PEPC2 had an eGFR below 90 mL/min/1.73 m^2^, and the proportion with albuminuria was very low at both PEPC1 and PEPC2 (7% and 5%). Thus, we did not analyze these outcomes as dichotomous variables in our regression models due to rare-event bias and anticipated imprecision in our effect size estimates. As our remaining primary outcomes were all measured on a continuous scale, we used an identity link function and normal distribution to estimate *β* with 95% confidence level (CL). We first estimated the associations of HDP with kidney function assessed in adolescence at PEPC1 with eGFR and then with urine ACR in separate models. Next, we estimated the associations of HDP with kidney function assessed in young adulthood at PEPC2 with eGFR and then with urine ACR in separate models. Secondarily, we estimated the associations of kidney function in adolescence at PEPC1 with kidney function in young adulthood at PEPC2 for both eGFR and urine ACR in separate models to confirm the expected within-participant correlation of kidney function over time. We initially performed complete case analyses.

**Fig. 2:**
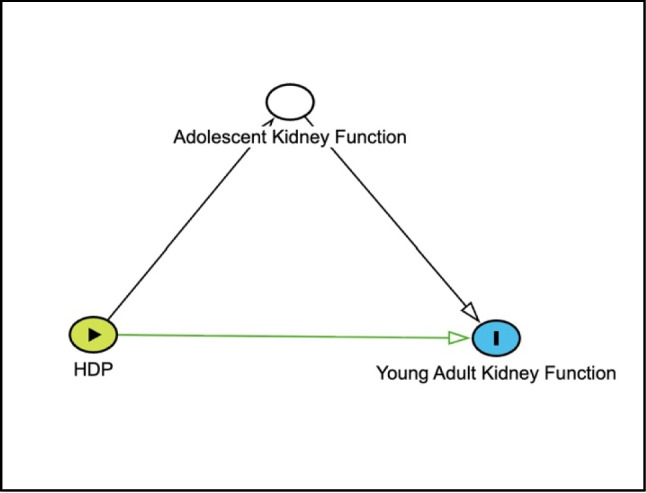
Directed acyclic graph (DAG) illustrating the direct effect of hypertensive disorders of pregnancy on young adult kidney function and the indirect effect mediated through adolescent kidney function. The green node is the exposure, HDP. The blue node is the outcome, young adult kidney function, and the white node is the mediator, adolescent kidney function. HDPs, hypertensive disorders of pregnancy

Applying a causal inference framework to our analytic plan, we developed graphical causal models of the relationships among our exposure, mediator, and outcomes using directed acyclic graphs (DAGs) a priori to inform the minimally sufficient adjustment sets included in each multivariable model to close biasing, non-causal paths (Fig. 2)[11, 12]. The DAGs were informed by the literature and our clinical and epidemiological expertise. Our adjustment sets included maternal smoking during pregnancy for the eGFR and urine ACR models at PEPC1 and PEPC2. For the model estimating the association of adolescent kidney function at PEPC1 with young adult kidney function at PEPC2, the adjustment sets included acute kidney injury in the neonatal intensive care unit, height at PEPC2 visit 1, and obesity at PEPC2 visit 1. The association of adolescent and young adult urine ACR was additionally adjusted for sex.

We performed a causal mediation analysis to estimate the direct effects of HDP (our exposure) on kidney function at PEPC2 (our outcome) and the indirect effects mediated by kidney function at PEPC1 (our mediator)[13]. We performed separate models for eGFR at PEPC1 and PEPC2 and then ACR at PEPC1 and PEPC2. We applied the following assumptions to our models, guided by our DAGs: (1) The effects of the exposure on each outcome were unconfounded conditional on our adjustment set; (2) the effect of the mediator on each outcome was unconfounded conditional on the adjustment set and on the exposure; (3) the effects of the exposure on each mediator were unconfounded conditional on the adjustment set; and (4) none of the mediator-outcome confounding factors were themselves affected by the exposure. We assessed for an interaction between the exposure and each mediator by adding an interaction term to the models and applied VanderWeele’s four-way effect decomposition method [14].

## Results

### Study population characteristics

Of the sample in this cohort, 193 were initially enrolled in PEPC1. Five were excluded for being a set of twins or having polycystic kidney disease, chromosome abnormality, and severe cerebral palsy. Additionally, three participants of that original PEPC1 group withdrew after their first visit. An additional 28 participants were added to the cohort for PEPC2. This made our participants for this study a total of 213 (Fig. 1).

Of the 213 included participants, 113 (53%) were female, 89 (42%) were Black, and 79 (37%) were exposed to HDP (Table 1). The mean eGFR was 130.2 mL/min/1.73 m^2^ at PEPC1 (*n* = 124) and 168.7 mL/min/1.73 m^2^ at PEPC2 (*n* = 159). The median ACR was 5.3 mg/g at PEPC1 and 3.1 mg/g at PEPC2 (Table 2). Kidney function in adolescence was associated with kidney function in young adulthood (eGFR adjusted *β* 0.48 mL/min/1.73 m^2^, 95% CL 0.21 to 0.75; ACR adjusted *β* 0.19 mg/g, 95% CL 0.15 to 0.24) (Table 2).

**Table 2:** Bivariate and multivariable associations of exposure to hypertensive disorders of pregnancy with offspring kidney function in adolescence and young adulthood

	Unadjusted *β* with 95% CL	Adjusted *β* with 95% CL	
HDP			
*PEPC1: adolescence*^*a*^			
eGFR (mL/min/1.73 m^2^), *n* = 124/123	−3.0 (−13.8 to 7.7)	−3.4 (−14.2 to 7.3)	
Urine ACR (mg/g), *n* = 137/136	−6.6 (−18.7 to 5.5)	−5.6 (−17.3 to 6.2)	
*PEPC2: young adulthood*^*b*^			Average causal mediation effect by adolescent kidney function
eGFR (mL/min/1.73 m^2^), *n* = 159/132	−1.8 (−9.0 to 5.4)	0.4 (−7.9 to 8.7)	−0.2 (−3.0, 2.7)
Urine ACR (mg/g), *n* = 157/133	0.8 (−5.3 to 6.9)	−0.5 (−7.6 to 6.6)	−1.4 (−4.9, 1.8)
Kidney function in adolescence			
One-unit higher eGFR (mL/min/1.73 m^2^) at PEPC2^c^, *n* = 89/63	**0.23 (0.01 to 0.44)**	**0.48 (0.21 to 0.75)**	
One-unit higher urine ACR (mg/g) at PEPC2^d^, *n* = 98/74	**0.2 (0.15 to 0.25)**	**0.19 (0.15 to 0.24)**	

*ACR* albumin-to-creatinine ratio, *eGFR* estimated glomerular filtration rate, *HDPs* hypertensive disorders of pregnancy, *PEPC* Prenatal Events-Postnatal Consequences. Individual model sample sizes are reported for each bivariate and multivariable model. Bold font indicates two-sided *p* < 0.05. Multivariable models include the following minimally sufficient adjustment sets

^a^HDP and adolescent kidney function at PEPC1: maternal smoking during pregnancy

^b^HDP and young adulthood kidney function at PEPC2: maternal smoking during pregnancy

^c^Adolescent kidney function at PEPC1 and young adult kidney function at PEPC2 (eGFR): acute kidney injury in the neonatal intensive care unit, height at PEPC2 V1, HDP, and obesity at PEPC2 V1 (n.b., age at PEPC2 V1 and sex were identified in the minimally sufficient adjustment set but are incorporated into the CKD-EPI eGFR equation and so were not additionally added as covariates)

^d^Adolescent kidney function at PEPC1 and young adult kidney function at PEPC2 (urine ACR): acute kidney injury in the neonatal intensive care unit, height at PEPC2 V2, HDP, obesity at PEPC2 V2, age at PEPC2 V2, and sex

### Association of HDP and kidney function at adolescence and young adulthood

There were no statistically significant associations between HDP and kidney function values in adolescence or young adulthood (Table 2). At PEPC1, the eGFR was 3.4 mL/min/1.73 m^2^ lower in the exposed group (adjusted 95% CL −14.2 to 7.3). The urine ACR at PEPC1 was 5.6 mg/g lower in the exposed group (adjusted 95% CL −17.3 to 6.2). At PEPC2, the eGFR was 0.4 mL/min/1.73 m^2^ higher in the exposed group (adjusted 95% CL −7.9 to 8.7). The urine ACR at PEPC2 was 0.5 mg/g lower in the exposed group (adjusted 95% CL −7.6 to 6.6).

### Causal mediation analysis

There was no statistically significant causal mediation effect from adolescent kidney function on young adulthood kidney function (Table 2). The average causal mediation effect by adolescent kidney function for eGFR was −0.2 mL/min/1.73 m^2^ (95% CL −3.0 to 2.7) and for urine ACR was −1.4 mg/g (95% CL −4.9 to 1.8).

## Discussion

In this analysis of long-term kidney function in young adults born preterm with very low or extremely low birth weight, exposure to HDP was not associated with offspring kidney function. Furthermore, kidney function in young adulthood was not mediated by earlier kidney function in adolescence. These findings are similar to those observed in Keijzer-Veen et al.’s prospective cross-sectional study done in 2007 in The Netherlands that examined young adult kidney function at 20 years of age who were born at < 32 weeks and who had small-for-gestational age birth weight (< −2 *SD*) (*n* = 23) or appropriate-for-gestational age birth weight (*n* = 29) [15]. They also did not find a difference in kidney function—assessed by inulin-based measured GFR—between exposure groups [15]. This study is relevant to our study as HDPs have been shown to be associated with growth restriction [16] and can negatively impact nephrogenesis [17]. There may be a greater risk of alterations to nephrogenesis during the third trimester, as preeclampsia has the highest incidence in the third trimester [17].

Previous research can provide possible explanations for the lack of association between HDP and offspring kidney function. For one, multiple other factors besides HDP can impact offspring kidney function, such as maternal body weight and maternal and offspring nutrition [18]. Lihme et al., in 2025, showed that offspring born term who were exposed to preeclampsia had a greater risk of all types of kidney disease (defined by ICD-10 codes in a national registry) compared to those who were unexposed except for acute kidney disease after 1 year of age. Since this was seen in the term-birth cohort, it indicates that other factors and mechanisms may be at play besides those associated with preterm birth, such as growth restriction and the associated negative impacts on nephrogenesis previously mentioned [17, 19]. Additionally, Wu et al. in 2024 demonstrated that kidneys in premature offspring can go through a period of catch-up growth where the nephrons are able to develop and allow the offspring’s kidneys to function normally by 3 years of age [20].

Previous studies that examined the effect of HDP on offspring outcomes found that there is a higher prevalence of adverse fetal outcomes when there are multiple HDP superimposed onto each other as opposed to one individually [21]. Bánhidy et al. in 2012 showed that there was a higher risk of renal dysgenesis in offspring with mothers who had preeclampsia superimposed on chronic hypertension as opposed to women who had the sole diagnosis of preeclampsia [22]. Yang et al. in 2022 demonstrated that there was a higher risk of adverse fetal outcomes in women with elevated blood pressure and superimposed preeclampsia, as opposed to women with only elevated blood pressure [21]. These studies reflect that there are multiple factors that can impact offspring outcomes and long-term kidney function, whereas our work specifically focused on the impact of HDP on kidney function as opposed to integrating other possible factors.

A strong point of this study is the well-characterized prospective longitudinal cohort with long-term follow-up that specifically recorded kidney function values at follow-up visits over several years. As previously mentioned, while other studies have examined the long-term impact of HDP on the function of other organ systems, few have specifically evaluated its association with kidney function long term. We also applied rigorous causal inference methods in our analytic plan to attempt to mitigate various sources and types of bias. However, a main limitation of this study is the lack of differentiation among the specific types of HDP. Those data were not reliably recorded with enough validity, and thus, we decided not to include them in this analysis due to the potential for measurement error leading to imprecision. Future research should investigate whether differences exist across the spectrum of HDP, especially for preeclampsia superimposed with chronic hypertension [23]. We did not collect additional follow-up urine samples to confirm an albuminuria diagnosis; future research should do so to be as accurate as possible. Furthermore, another limitation is that this project was a secondary analysis of data from the original PEPC1 study and thus was not powered to specifically answer this question. Lastly, the addition of new participants added during the PEPC2 phase who were not included in PEPC1 but were eligible is another limitation as we did not have a full dataset for these participants, thereby limiting the longitudinal modeling.

Future research needs to continue to investigate if differences in kidney function manifest in older adulthood as this cohort and others age, as differences in kidney function and risk of chronic kidney disease may manifest in mid to older adulthood. In addition, differentiating between the different HDP will be important to evaluate their individual impact on the offspring’s kidney function. As those disorders have different pathophysiologic mechanisms for how they affect the fetus, it is essential to evaluate their effects separately [21]. Future research should consider investigating what role preterm versus term birth and lower birth weight versus normal or high birth weight play in the association with the change in kidney function and development of chronic kidney disease over the life course and to what extent HDP may be responsible mechanistically [19]. Furthermore, it has been demonstrated that neonates born at earlier stages of preterm gestation are at higher risk for more health complications later in life [24]. It would, therefore, be justified to examine if the weeks of gestation at delivery had an association with adolescent and young adult kidney function.

## Conclusion

We did not observe differences in kidney function during adolescence or early adulthood in individuals born preterm with very low or extremely low birth weight who were exposed to HDP. Additionally, we did not find that adolescent kidney function mediated the association between HDP and young adult kidney function. Future research should continue to investigate the change in kidney function into middle and older adulthood among affected individuals who may be at higher risk of chronic kidney disease.

## Supplementary Information

Below is the link to the electronic supplementary material.

Graphical abstract (PPTX 113 KB)

## Data Availability

Data from this analysis are available upon reasonable request.

## Declarations

### Previous presentations

Portions of these results were presented in abstract form at ASN Kidney Week, October 24–27, 2024, San Diego, CA.

## References

[1] (2013) Hypertension in pregnancy. Report of the American College of Obstetricians and Gynecologists’ Task Force on Hypertension in Pregnancy. Obstet Gynecol 122:1122–1131. 10.1097/01.aog.0000437382.03963.88 10.1097/01.AOG.0000437382.03963.88 24150027

[2] Jones DW, Ferdinand KC, Taler SJ, Johnson HM, Shimbo D, Abdalla M, Altieri MM, Bansal N, Bello NA (2025) 2025 AHA/ACC/AANP/AAPA/ABC/ACCP/ACPM/AGS/AMA/ASPC/NMA/PCNA/SGIM guideline for the prevention, detection, evaluation and management of high blood pressure in adults: a report of the American College of Cardiology/American Heart Association Joint Committee on Clinical Practice Guidelines. Circulation 152:e114–e218. 10.1161/CIR.0000000000001356 40811497 10.1161/CIR.0000000000001356

[3] Barker DJ, Osmond C, Golding J, Kuh D, Wadsworth ME (1989) Growth in utero, blood pressure in childhood and adult life, and mortality from cardiovascular disease. BMJ 298:564–567. 10.1136/bmj.298.6673.564 2495113 10.1136/bmj.298.6673.564 PMC1835925

[4] Ohuma EO, Moller A-B, Bradley E, Chakwera S, Hussain-Alkhateeb L, Lewin A, Okwaraji YB, Mahanani WR, Johansson EW, Lavin T, Fernandez DE, Domínguez GG, De Costa A, Cresswell JA, Krasevec J, Lawn JE, Blencowe H, Requejo J, Moran AC (2023) National, regional, and global estimates of preterm birth in 2020, with trends from 2010: a systematic analysis. Lancet 402:1261–1271. 10.1016/S0140-6736(23)00878-4 37805217 10.1016/S0140-6736(23)00878-4

[5] South AM, Nixon PA, Chappell MC, Diz DI, Russell GB, Jensen ET, Shaltout HA, O’Shea TM, Washburn LK (2019) Renal function and blood pressure are altered in adolescents born preterm. Pediatr Nephrol 34:137–144. 10.1007/s00467-018-4050-z 30112655 10.1007/s00467-018-4050-z PMC6237649

[6] Battaglia FC, Lubchenco LO (1967) A practical classification of newborn infants by weight and gestational age. J Pediatr 71:159–163. 10.1016/S0022-3476(67)80066-0 6029463 10.1016/s0022-3476(67)80066-0

[7] Oken E, Kleinman KP, Rich-Edwards J, Gillman MW (2003) A nearly continuous measure of birth weight for gestational age using a United States national reference. BMC Pediatr 3:6. 10.1186/1471-2431-3-6 12848901 10.1186/1471-2431-3-6 PMC169185

[8] Floyd WN, Beavers DP, Jensen ET, Washburn LK, South AM (2023) Association of antenatal corticosteroids with kidney function in adolescents born preterm with very low birth weight. J Perinatol 43:1038–1044. 10.1038/s41372-023-01688-3 37160975 10.1038/s41372-023-01688-3 PMC10524661

[9] South AM, Shaltout HA, Gwathmey TM, Jensen ET, Nixon PA, Diz DI, Chappell MC, Washburn LK (2020) Lower urinary α-Klotho is associated with lower angiotensin-(1–7) and higher blood pressure in young adults born preterm with very low birthweight. J Clin Hypertens 22:1033–1040. 10.1111/jch.13897 10.1111/jch.13897 PMC8030001 32475043

[10] South AM, Shaltout HA, Nixon PA, Diz DI, Jensen ET, O’Shea TM, Chappell MC, Washburn LK (2020) Association of circulating uric acid and angiotensin-(1-7) in relation to higher blood pressure in adolescents and the influence of preterm birth. J Hum Hypertens 34:818–825. 10.1038/s41371-020-0335-3 32346123 10.1038/s41371-020-0335-3 PMC7606311

[11] Cohen JB, D’Agostino McGowan L, Jensen ET, Rigdon J, South AM (2021) Evaluating sources of bias in observational studies of angiotensin-converting enzyme inhibitor/angiotensin II receptor blocker use during COVID-19: beyond confounding. J Hypertens 39:795–805. 10.1097/hjh.0000000000002706 33186321 10.1097/HJH.0000000000002706 PMC8164085

[12] Textor J, Hardt J, Knuppel S (2011) DAGitty: a graphical tool for analyzing causal diagrams. Epidemiology 22:745. 10.1097/ede.0b013e318225c2be 21811114 10.1097/EDE.0b013e318225c2be

[13] Zhang Z, Zheng C, Kim C, Van Poucke S, Lin S, Lan P (2016) Causal mediation analysis in the context of clinical research. Ann Transl Med 4:425. 10.21037/atm.2016.11.11 27942516 10.21037/atm.2016.11.11 PMC5124624

[14] VanderWeele TJ (2014) A unification of mediation and interaction: a 4-way decomposition. Epidemiology 25:749–761. 10.1097/ede.0000000000000121 25000145 10.1097/EDE.0000000000000121 PMC4220271

[15] Keijzer-Veen MG, Kleinveld HA, Lequin MH, Dekker FW, Nauta J, de Rijke YB, van der Heijden BJ (2007) Renal function and size at young adult age after intrauterine growth restriction and very premature birth. Am J Kidney Dis 50:542–551. 10.1053/j.ajkd.2007.06.015 17900453 10.1053/j.ajkd.2007.06.015

[16] Mecacci F, Romani E, Clemenza S, Zullino S, Avagliano L, Petraglia F (2024) Early fetal growth restriction with or without hypertensive disorders: a clinical overview. Reprod Sci 31:591–602. 10.1007/s43032-023-01330-9 37684516 10.1007/s43032-023-01330-9

[17] Koulouraki S, Paschos V, Pervanidou P, Christopoulos P, Gerede A, Eleftheriades M (2023) Short- and long-term outcomes of preeclampsia in offspring: review of the literature. Children 10:826. 10.3390/children10050826 37238374 10.3390/children10050826 PMC10216976

[18] Raju TNK, Buist AS, Blaisdell CJ, Moxey-Mims M, Saigal S (2017) Adults born preterm: a review of general health and system-specific outcomes. Acta Paediatr 106:1409–1437. 10.1111/apa.13880 28419544 10.1111/apa.13880

[19] Lihme I, Basit S, Lihme F, Damholt MB, Hjorth S, Nohr EA, Boyd HA (2025) A nationwide register-based cohort study examined the association between preeclampsia in mothers and the risk of kidney disease in their offspring. Kidney Int 108:283–292. 10.1016/j.kint.2025.04.017 40383228 10.1016/j.kint.2025.04.017

[20] Wu Y, Allegaert K, Flint RB, Goulooze SC, Valitalo PAJ, de Hoog M, Mulla H, Sherwin CMT, Simons SHP, Krekels EHJ, Knibbe CAJ, Voller S (2024) When will the glomerular filtration rate in former preterm neonates catch up with their term peers? Pharm Res 41:637–649. 10.1007/s11095-024-03677-3 38472610 10.1007/s11095-024-03677-3 PMC11024008

[21] Yang Y, Xie Y, Li M, Mu Y, Chen P, Liu Z, Wang Y, Li Q, Li X, Dai L, Liang J, Zhu J (2022) Characteristics and fetal outcomes of pregnant women with hypertensive disorders in China: a 9-year national hospital-based cohort study. BMC Pregnancy Childbirth 22:924. 10.1186/s12884-022-05260-3 36482386 10.1186/s12884-022-05260-3 PMC9733350

[22] Bánhidy F, Szilasi M, Czeizel AE (2012) Association of pre-eclampsia with or without superimposed chronic hypertension in pregnant women with the risk of congenital abnormalities in their offspring: a population-based case–control study. Eur J Obstet Gynecol Reprod Biol 163:17–21. 10.1016/j.ejogrb.2012.03.015 22504080 10.1016/j.ejogrb.2012.03.015

[23] Wu CS, Nohr EA, Bech BH, Vestergaard M, Catov JM, Olsen J (2009) Health of children born to mothers who had preeclampsia: a population-based cohort study. Am J Obstet Gynecol 201:269.e261-269.e210. 10.1016/j.ajog.2009.06.060 10.1016/j.ajog.2009.06.060 19733276

[24] de Gamarra-Oca LF, Ojeda N, Gómez-Gastiasoro A, Peña J, Ibarretxe-Bilbao N, García-Guerrero MA, Loureiro B, Zubiaurre-Elorza L (2021) Long-term neurodevelopmental outcomes after moderate and late preterm birth: a systematic review. J Pediatr 237:168–176. 10.1016/j.jpeds.2021.06.004 34171360 10.1016/j.jpeds.2021.06.004

